# Deep Learning for Sensor-Based Rehabilitation Exercise Recognition and Evaluation†

**DOI:** 10.3390/s19040887

**Published:** 2019-02-20

**Authors:** Zheng-An Zhu, Yun-Chung Lu, Chih-Hsiang You, Chen-Kuo Chiang

**Affiliations:** Advanced Institute of Manufacturing with High-tech Innovations, Center for Innovative Research on Aging Society (CIRAS) and Department of Computer Science and Information Engineering, National Chung Cheng University, Chiayi 62102, Taiwan; cca104m@cs.ccu.edu.tw (Z.-A.Z.); lyc105m@cs.ccu.edu.tw (Y.-C.L.); ych103m@cs.ccu.edu.tw (C.-H.Y.)

**Keywords:** rehabilitation exercises, recognition, evaluation, deep learning, sensor data

## Abstract

In this paper, a multipath convolutional neural network (MP-CNN) is proposed for rehabilitation exercise recognition using sensor data. It consists of two novel components: a dynamic convolutional neural network (D-CNN) and a state transition probability CNN (S-CNN). In the D-CNN, Gaussian mixture models (GMMs) are exploited to capture the distribution of sensor data for the body movements of the physical rehabilitation exercises. Then, the input signals and the GMMs are screened into different segments. These form multiple paths in the CNN. The S-CNN uses a modified Lempel–Ziv–Welch (LZW) algorithm to extract the transition probabilities of hidden states as discriminate features of different movements. Then, the D-CNN and the S-CNN are combined to build the MP-CNN. To evaluate the rehabilitation exercise, a special evaluation matrix is proposed along with the deep learning classifier to learn the general feature representation for each class of rehabilitation exercise at different levels. Then, for any rehabilitation exercise, it can be classified by the deep learning model and compared to the learned best features. The distance to the best feature is used as the score for the evaluation. We demonstrate our method with our collected dataset and several activity recognition datasets. The classification results are superior when compared to those obtained using other deep learning models, and the evaluation scores are effective for practical applications.

## 1. Introduction

Rehabilitation exercise is one of the most important steps for recovery after surgery, especially after joint disease surgery. A home exercise program is common for rehabilitation treatment where a patient performs a set of physical exercises in a home-based environment. However, such exercises are not always successful in helping the patients reach full recovery. One of the main barriers is that patients do not comply with the prescribed exercise plans. In addition, this program lacks supervision and the monitoring of patient performance.

In the computer vision and machine learning fields, action recognition has received increasing attention. Activity recognition can be classified into two categories, namely, vision-based and sensor-based methods. For vision-based methods, human actions can be viewed as a set of spatio-temporal changes of appearances or motions. Methods devoted to effective visual representation for action recognition in videos or still images include shape-based movement analysis [1], temporal templates [2], and space–time volume [3]. Although many vision-based methods have been used over the past decades, large variations in human pose, occlusion, and viewpoint change still make this problem very challenging. Sensor technologies, especially low-power, high-capacity, wireless communication, and data processing, have made substantial progress, making it possible for sensors to evolve from low-level data collection and transmission to high-level inference. Wearable sensors can be embedded into clothes, belts, smart watches, and mobile devices for information collection and analysis. Rehabilitation movement is composed of a series of actions. In contrast to action recognition which recognizes the action as different classes, action evaluation aims to score the action. This is particularly important to rehabilitation because it indicates if the patient can complete the action or not. Moreover, the score implies the level of recovery from a particular injury.

In this paper, we aim to propose sensor-based action recognition and evaluation models. One challenge using sensor data for activity recognition is the data alignment problem. The start and end time, as well as the speed, required to perform activities may be totally different. The data also contain noises and variations when the activity is performed by different persons. To overcome this issue, a dynamic convolutional neural network (D-CNN) is proposed. In addition, in order to capture the hidden states of sensor data, a state transition probability convolutional neural network (S-CNN) is proposed for feature representation by the transition probabilities between states. Then, a Multipath convolutional neural network (MP-CNN) is constructed to recognize the class of rehabilitation exercise. To evaluate the rehabilitation exercises, we propose to use a specially designed matrix along with the learned classifier to infer the best feature of each class at different levels.

The contribution of our method is three-fold. First, we propose a new multipath deep learning model for rehabilitation exercise classification. The dynamic CNN (D-CNN) is extended by combining it with a state transition probability CNN (S-CNN) to overcome the data alignment problem and find the hidden states of exercise for discriminative feature representation. Then, a novel evaluation method is proposed by learning the best feature of each class. When the current exercise is classified, the feature can be extracted. The evaluation score is obtained by examining the distance measure of the current feature and the best feature of that class. We also collect a new rehabilitation exercise dataset for the rehabilitation exercise evaluation. It contains four different rehabilitation actions at three levels, defined by rehabilitation physicians. More details about our dataset can be found in Section 5.1. Experimental results on our collected dataset and several activity recognition datasets demonstrate the superior performance of the proposed model.

The rest of this paper is organized as follows. The literature reviews are introduced in Section 2. Our action recognition method is presented in Section 3, including the state transition probability CNN (S-CNN), the dynamic CNN (D-CNN), and the multipath CNN (MP-CNN). In Section 4, the rehabilitation exercise evaluation model is introduced. Our collected dataset and the experimental results are presented in Section 5. Discussion about the properties and problems of the approach is given in Section 6. Finally, we conclude this paper in Section 7.

## 2. Related Work

With the recent advances in wearable devices, human activity recognition can be achieved by collecting sensor data via such devices. Activity recognition plays an important role in daily life and has a significant impact on many applications, such as daily lifelog [4], health care [5], elderly care [6], and personal fitness [7]. Sensor-based human activity recognition has become an important issue in recent years. Action recognition can be divided into three parts: preprocessing, feature extraction, and posture recognition. In early research studies, sensor acquisition devices were limited and used mainly for the development of new features and classifiers. To process sensor data, feature extraction is usually adopted as the first step. Traditionally, it captures statistical information through the mean, variance, or entropy to extract features [8,9,10]. Other statistical methods in the frequency domain, such as Fast Fourier Transform (FFT) [11], are also widely used. However, these methods are applicable to single-action identification and ineffective for the recognition of multiple actions [12]. Principal component analysis (PCA) is a common technique used to capture sensor data features. Since it can only capture the linear structure of feature space, nonlinear methods, such as support vector machine (SVM) [13], hidden Markov models (HMMs) [14], etc., are required for complicated activities.

Representative human activity recognition methods can be classified into two categories, namely, heuristic methods and template matching methods. The heuristic approach depends on knowledge and is related to specific tasks. Reyes-Ortiz et al. [15] recognized actions and postural transitions by designing temporal filters. Template matching methods are often derived from the Longest Common Subsequence (LCSS) [16,17] and dynamic time warping (DTW) [18] methods. WarpingLCSS and SegmentedLCSS were proposed by improving the LCSS algorithm, which were more robust to noisy annotation. Hartmann and Link [18] constructed a segmented DTW method to bound and recognize a gesture in each frame by finding the best matching among the objects as well as all templates of different classes. For sensor-based action recognition, accelerometers are probably the most commonly used wearable devices. They are effective in repetitive actions, such as running, walking, sitting, and standing. In [19], a network of three-axis accelerometers was positioned over the user’s body. It provided the orientation and movement of the body part. Subsequently, Lukowicz et al. [20] recognized activities by measuring acceleration and angular velocity through accelerometers and gyroscopes. Lee and Mase [21] proposed a dead-reckoning method to calculate a user’s location for behavior recognition.

For rehabilitation action recognition, Kinect is used for analyzing depth images. According to the key points of the human body, vector angle and vector modulus ratio are combined for body feature representation. Then, a dynamic time warping (DTW) algorithm is applied for action matching [22]. In [23], support vector machines (SVMs) and random forests (RF) were introduced on the PCA feature space to accurately classify Kinect’s kinematic activities. Global descriptors [24] of the dynamical attractor’s shape were proposed as features for modeling actions. It outperformed kinematic analysis and chaotic invariant-based methods in the estimation of movement quality. Many studies based on deep learning have been conducted recently. These works are mainly derived from convolutional neural networks (CNNs) and recurrent neural networks (RNNs) [25,26,27,28]. The CNN is one of the most popular methods in deep learning [29,30]. Features extracted by the CNN from sensor signals for activity recognition have two advantages. The CNN can capture local dependency of signals and also process signals in different scales, such as frequency or amplitude. In this paper, we propose a rehabilitation action evaluation and classification model based on the CNN technique.

## 3. Sensor-Based Rehabilitation Exercise Recognition

Rehabilitation movement consists of one or more time series actions. For example, the user may raise his hands first with fingers crossed and then bend over to the right of the body. Other movements may contain only one action, such as move shoulders upward. In this paper, we propose a multipath CNN (MP-CNN) to capture local dependency of activity signals and classify the action class based on our previous work [31]. The MP-CNN consists of two sub-networks: a dynamic convolutional neural network (D-CNN) and a state transition probability CNN (S-CNN). We also propose a new evaluation method to score the rehabilitation exercise based on the classifier learning of deep models. We will explain each component in the sub-sections.

### 3.1. State Transition Probability CNN (S-CNN)

Conventionally, features can be represented by the transition probabilities between states using the probabilistic finite state automata (PFSA) [32]. However, the computational complexity of calculating the transition probabilities is high. Therefore, we propose to use the CNN to model the relations between input signals and the transition probabilities between states for more discriminative feature representation.

**Figure 1:**
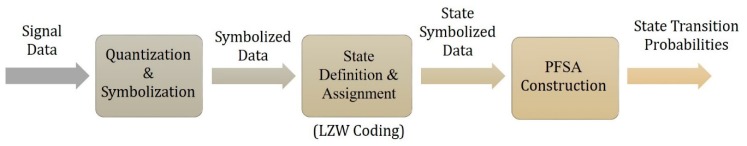
Flowchart of the Lempel–Ziv–Welch (LZW)-coded probabilistic finite state automata (PFSA) method.

The Lempel–Ziv–Welch (LZW)-coded PFSA [32] uses the LZW coding to symbolize sensor data and the PFSA to compute the state transition probabilities between hidden states. It consists of three steps: quantization, LZW coding, and PFSA construction. The flowchart is shown in Figure 1. We built an S-CNN to model the transition probabilities in the LZW-coded PFSA [32] to extract discriminative features. We explain the LZW-coded PFSA method first and then how to combine the state transition probabilities into the CNN model as an S-CNN.

#### 3.1.1. Quantization

All training signals are first concatenated into a single vector and then sorted in ascending order. The vector is then divided into *K* parts to represent *K* levels. The boundary of each part denotes the level boundary. Next, the raw data are symbolized into the level index by each level boundary to reduce the complexity of processing the raw data.

#### 3.1.2. Flowchart of the Lempel-Ziv-Welch (LZW) Coding

The LZW algorithm first computes the LZW table and uses the table to encode the sequence. In the first step, the input stream is *aaab* and it is used to initialize the coding table. In the second step, *P* is *a*, *C* is *a*, and *P* + *C* is not in the table from the first step. Thus, it is added into the table and sets *P* = *C*. In the third step, *P* + *C* is in the table from the second step. Therefore, *P* = *P* + *C* is *aa*. In addition, *C* is *b* and *P* + *C* is not in the table from the third loop. It is added into the table and *P* = *C*. In the fourth step, the stream is empty, so the loop is stopped. In the fifth step, it starts to encode the string. The stream is initialized. In the sixth step, *P* is *a*, *C* is *a*, and *P* + *C* is in the table from the first loop. Therefore, *P* = *P* + *C* is *aa*. In addition, *C* is *a* and *P* + *C* is not in the table from the second loop, so *P* is encoded and then *P* = *C*. In the seventh step, *C* is *b* and *P* + *C* is not in the table from the third loop, so *P* is encoded and then *P* = *C*. In the eighth step, the stream is empty, so the loop is stopped and then *P* is encoded. The symbolized data encoded by a modified LZW is shown in Algorithm 1. In order to reduce the number of states, *C* states are chosen in each class that occurs most frequently to compose a state dictionary *D*. Then, the dictionary is used to quantize the state which is not in the dictionary *D* by computing the Levenshtein distance between the state and each state in *D*. The quantization can be applied as (1):

(1) $$S_{map}\left(s_{u}\right)=min_{s_{k},\;k\in D}\;lev_{s_{u},s_{k}}\left(\left|s_{u}\right|,\left|s_{k}\right|\right),$$

where $s_{u}$ is the state not in the dictionary *D*,$\;s_{k}$ is the state in the dictionary *D*, $lev_{s_{u},s_{k}}\left(.\right)$ is the operator to calculate the Levenshtein distance, $\left|s_{u}\right|$ is the string of the $s_{u}$ state, $\left|s_{k}\right|$ is the string of the $s_{k}$ state, and $S_{map}$ is the mapping function to quantize the $s_{u}$ into $s_{k}$.

#### 3.1.3. PFSA Construction

The PFSA is used to record the state transition probabilities of the state symbolized data from LZW coding. The PFSA is a state diagram which records the transition probabilities between each state. The state transition probabilities can be computed as (2):

(2) $$P\left(o_{i}|o_{j}\right)=\frac{N\left(o_{j},\;o_{i}\right)}{\sum_{i=1}^{n}N\left(o_{j},\;o_{i}\right)}\;\forall o_{i},\;o_{j}\in O,\;1\leq i,j\leq n,$$

where *O* is the set of states, *n* is the number of states, and $N(o_{j},\;o_{i}$) is the number of transitions from $o_{j}$ to $o_{i}$. Therefore, the state transition probabilities can be represented by a matrix π as (3):

(3) $$\pi =\left[\begin{array}{ccc} P\left(o_{1}|o_{1}\right) & \cdots & P\left(o_{n}|o_{1}\right)\\ \vdots & \ddots & \vdots \\ P\left(o_{1}|o_{n}\right) & \cdots & P\left(o_{n}|o_{n}\right) \end{array}\right].$$

**Algorithm 1:** Modified LZW encoding algorithm
**1**
**Task 1**: Encoding the sequence *S* and finding the LZW table *T*
**2**
**Initialization**: Initializing table *T* with single character in the sequence *S*
**3**
**Output**: code and table *T*
**4**
Set *P* = first input character in *S*
**5**
**while** not end of the sequence *S*
**6**
  *C* = next input character in *S*
**7**
  **if**
*P* + *C* is in the table *T*
**8**
    *P* = *P* + *C*
**9**
  **else**
**10**
    add *P* + *C* to the table *T*
**11**
    *P* = *C*
**12**
  **end if**
**13**

**end while**

**14**
Set *P* = first input character in *S*
**15**
**while** not end of the sequence *S*
**16**
  *C* = next input character in *S*
**17**
  **if**
*P* + *C* is in the table *T*
**18**
    *P* = *P* + *C*
**19**
  **else**
**20**
    output the code for *P*
**21**
    *P* = *C*
**22**
  end if
**23**

**end while**

**24**

**output code for P**


#### 3.1.4. S-CNN Model

The state transition matrix π from Section 3.1.3 is formed into a vector as the output (ground truth) of the S-CNN model. The raw data are used as input to train the S-CNN with two layers of both convolution and pooling. Then, the results obtained by the last layer of pooling are used as input to the fully connected layers. The S-CNN model can be viewed as a regression model to map sensor signals to the state transient probabilities learned by the LZW-coded PFSA in order to find discriminative features for classification. 

The input signals of our method are retrieved from three-axis accelerometers and used for the S-CNN to obtain the transition probabilities. To train the second component, the D-CNN, features are extracted from the raw data. To preprocess the raw data, a fixed-size sliding window is applied. Then, a median filter (size: 3) is used to remove the signal noises. The signals are then normalized to the range [0, 1]. To extract features from input signals, a low-pass filter [33,34] is applied to extract the gravity and body acceleration as two features of the three-axis signals. The gravity feature is the signal associated with the gravitational acceleration. The signals without gravitational acceleration correspond to the body feature.

### 3.2. Dynamic CNN (D-CNN)

One of the challenges using sensor data for activity recognition is the data alignment problem. In practical applications, the start and end time of activities may be different. The data also contain noises or variations when the activity is performed by different persons. Therefore, a dynamic CNN is proposed based on the construction of a Gaussian mixture model–Gaussian mixture regression (GMM-GMR) model. The GMM is learned for each activity as the comparison standard. During testing, the proposed dynamic assignment method is applied via two operations, namely, data partition and channel fitting, to fit the data and the GMM. Then, the matched data are used as the input into the corresponding channel in the CNN model.

#### 3.2.1. Gaussian mixture model–Gaussian mixture regression (GMM-GMR) Model

GMM-GMR [35] is used to model the distributions of different activities. This overcomes the within-class variations when collecting sensor data for one activity. We define $g_{t}=\left(t,\;g_{x}\left(t\right),g_{y}\left(t\right),\;g_{z}\left(t\right)\right)$, $g_{t}\in R^{4},\;t=1,\ldots ,T$, where time size is *T* and $b_{t}=\left(t,\;b_{x}\left(t\right),b_{y}\left(t\right),\;b_{z}\left(t\right)\right)$, $b_{t}\in R^{4},\;t=1,\ldots ,T$. GMM with *K* components are used to model $g_{t}$ and $b_{t}$. We define $\mu_{k}\in R^{4}$ and $\sum_{k}\in R^{4\times 4}$ as the mean vector and the covariance matrix, and for $g_{t}$, where *k* = 1, …, *K*. We use GMR to determine the mean and the covariance matrix at time *t* for the *k*-*th* GMM component. We first separate the temporal and acceleration values in $\mu_{k}$ and $\sum_{k}$ as (4):

(4) $$\mu_{k}=\left\{\mu_{k}^{t},\;\mu_{k}^{a}\right\}\sum_{k}=\left(\begin{array}{cc} \sum_{k}^{tt} & \sum_{k}^{ta}\\ \sum_{k}^{at} & \sum_{k}^{aa} \end{array}\right).$$

The expected mean acceleration ${\hat{\mu }}_{k}^{a}$ of the *k* component at time index *t* and the associated covariance matrix ${\hat{\sum }}_{k}^{aa}$ can be defined as (5):

(5) $$\{\begin{array}{c} {\hat{\mu }}_{k}^{a}=\mu_{k}^{a}+\sum_{k}^{at}{\left(\sum_{k}^{tt}\right)}^{-1}\left(t-\mu_{k}^{t}\right)\\ {\hat{\sum }}_{k}^{aa}=\sum_{k}^{aa}-\sum_{k}^{at}{\left(\sum_{k}^{tt}\right)}^{-1}\sum_{k}^{ta} \end{array}.$$

**Figure 2:**
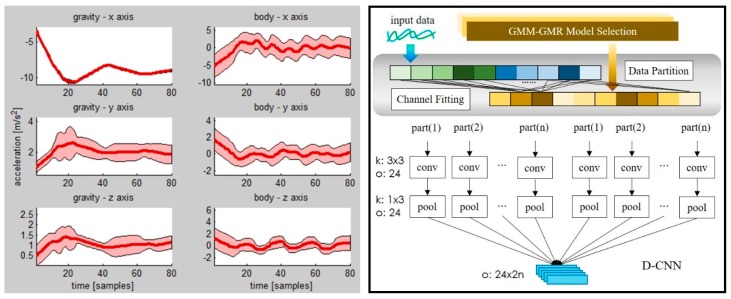
Left: Gaussian mixture model–Gaussian mixture regression (GMM-GMR) model of the “climb stairs” activity in the wearable human activity recognition folder (WHARF) dataset. Red line is acceleration value of every time point. Pink area is standard deviation of every time point. Right: Dynamic convolutional neural network (CNN) model. *k* is kernel size. o is the number of the output feature map.

Then, the ${\hat{\mu }}_{k}^{a}$ and ${\hat{\sum }}_{k}^{aa}$ are mixed by the probability $\beta_{k}$ of the *k* component at time index *t* to compute the expected acceleration $\mu^{a}$ and covariance matrix $\sum^{aa}$ at time index *t*, as follows:

(6) $$\beta_{k}=\frac{p\left(k\right)p(t|k)}{\sum_{j=1}^{K}p\left(j\right)p(t|j)}=\frac{\pi_{k}N\left(t;\mu_{k}^{t},\sum_{k}^{tt}\right)}{\sum_{j=1}^{K}\pi_{j}N\left(t;\mu_{j}^{t},\sum_{j}^{tt}\right)},$$

(7) $$\mu^{a}=\sum_{k=1}^{K}\beta_{k}{\hat{\mu }}_{k}^{a}\;\sum^{aa}=\overset{K}{\underset{k=1}{\sum }}\beta_{k}^{2}{\hat{\sum }}_{k}^{aa}\;,$$

where N is the Gaussian distribution function. Therefore, we can calculate the mean and the covariance matrix of acceleration at every time *t* in the sequence *T* to build the GMM-GMR model, as shown in Figure 2.

#### 3.2.2. Dynamic Assignment

Once the GMM-GMR model is trained, the dynamic assignment approach is adopted to fit the input signal to the GMM-GMR model in a segment basis. The dynamic assignment contains two steps: data partition and channel fitting. In data partition. The GMM-GMR model is trained for each activity class. Then, the model is partitioned into *N* parts which correspond to the *N* channels in the D-CNN. The features are also partitioned into *N* parts. By channel fitting, features which are similar to the same model part go to the same channel in the D-CNN. In channel fitting, the distance of partitioned features and the model part can be calculated by the Mahalanobis distance. Denoting $x_{t}$ as the triaxial acceleration signal at time *t*, the Mahalanobis distance between the signal at time *t* and the model part can be defined as (8):

(8) $$d_{t}=\sqrt{{\left(x_{t}-\mu_{t}^{a}\right)}^{T}{\left(\sum_{t}^{aa}\right)}^{-1}\left(x_{t}-\mu_{t}^{a}\right)},$$

where $\mu_{t}^{a}$ and $\sum_{t}^{aa}$ are the mean and the covariance matrix of the model at time *t*. Therefore, the distance between the partitioned features and the model part can be computed as (9):

(9) $$d=\frac{1}{n}\sum_{t=1}^{n}d_{t},$$

where *n* is the size of the partitioned feature.

The *N* model parts correspond to *N* channels of the CNN. Here, channel is referred to as one path containing convolution and pooling operations in the CNN. Then, the results of the *N* channels are concatenated to build the feature map. The D-CNN’s process is depicted in Figure 2.

### 3.3. Sensor-Based Rehabilitation Exercise Recognition by the Multipath CNN (MP-CNN)

**Figure 3:**
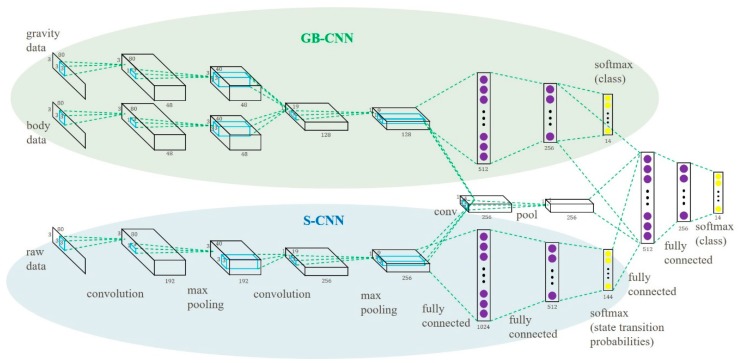
Multipath CNN model.

The proposed multipath CNN (MP-CNN) consists of two CNN models, a top CNN model and a bottom CNN model, to improve the recognition accuracy. First, we propose a CNN model using the Gravity and Body (GB) signals as input, called the GB-CNN. In the GB-CNN model, the gravity path and the body path are combined for common convolution and pooling operations to capture joint features. The outputs are used as feature input into two hidden layers. The GB-CNN model is depicted at the top of Figure 3. And the S-CNN model is depicted at the bottom of Figure 3.

**Figure 4:**
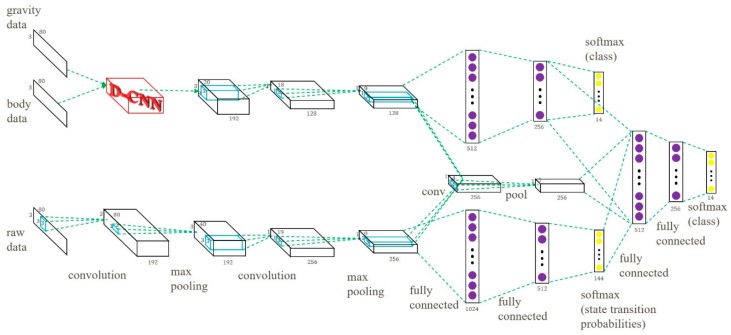
Multipath CNN model with D-CNN.

We combined the GB-CNN and the S-CNN models and built the MP-CNN. In the MP-CNN, the results of the second pooling layer in the two models were combined and convolution and pooling operations were applied again to capture the data correlation between the features of the GB-CNN and the S-CNN. Then, the outputs were combined with the results of the second hidden layer in the GB-CNN and the output layer in the S-CNN and were used as input into another two hidden layers. In order to improve the performance of the MP-CNN, the D-CNN model was included. In the MP-CNN, the gravity and body features were used as input into the D-CNN model. Finally, the feature maps from the D-CNN were used in convolution and pooling operations to capture the correlation between the two features. The whole MP-CNN model is depicted in Figure 4. The process of the learning MP-CNN is summarized in Algorithm 2.

**Algorithm 2:** Multipath Convolutional Neural Network
**1**
**Task 1**: Learning S-CNN model
**2**
**Input**: Raw sensor signals
**3**
**Output**: State transition probabilities
**4**
Step 1: Quantization
**5**
Step 2: Symbolization
**6**
Step 3: LZW coding
**7**
Step 4: PFSA construction
**8**
Step 5: Obtain state transition probabilities
**9**
Step 6: S-CNN model training
**10**

**End**

**11**


**12**
**Task 2**: Learning D-CNN model
**13**
**Input**: Raw sensor signals
**14**
**Output**: Classification results
**15**
Step 1: Feature extraction, gravity and body features
**16**
Step 2: GMM-GMR model learning
**17**
Step 3: Data partition and channel fitting
**18**
Step 4: D-CNN model learning
**19**

**End**

**20**


**21**
**Task 3**: Learning MP-CNN
**22**
**Input**: Raw sensor signals
**23**
**Output**: Classification results
**24**
Step 1: Model setup (as depicted in Figure 4)
**25**
Step 2: Exploiting S-CNN and D-CNN as pre-train weights
**26**
Step 3: MP-CNN training
**27**

**End**


## 4. Sensor-Based Rehabilitation Exercise Evaluation

**Figure 5:**
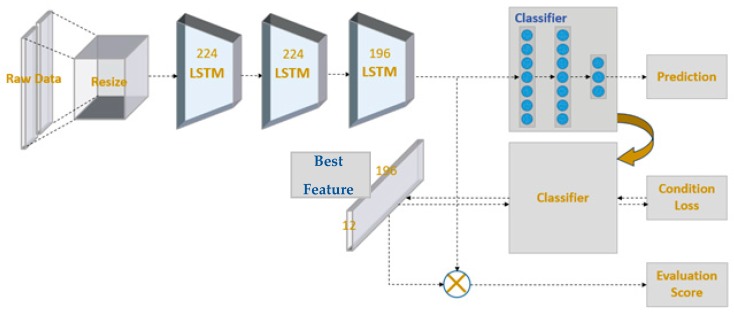
Model architecture for rehabilitation evaluation.

If an MP-CNN classifier can learn, it will be beneficial for rehabilitation exercise evaluation. We aimed to evaluate four kinds of rehabilitation exercises at three levels: good, average, and bad. The idea was to design an evaluation matrix where each entry corresponded to one level of one exercise. By setting the largest number in one entry, the evaluation matrix could be used along with the output layer of the deep learning model to infer the best feature of that exercise at a particular level. For example, taking four kinds of rehabilitation exercises at three levels, our model found twelve best features for exercises at different levels. As a result, when one is performing the exercise, the feature can be extracted and compared with the best feature to evaluate the score of that exercise. This can be beneficial for users who need to correct their movement during rehabilitation. To simplify the explanation of the evaluation method, we used a simple long short-term memory (LSTM) [36] model as the classifier in this section. In our evaluation model, we used prediction loss, condition loss, and evaluation loss to learn the model weights. They were also the three output layers of our model. The system architecture is depicted in Figure 5. The details of these functions will be discussed later in this section.

### 4.1. Prediction Loss

We labeled the data as several categories, which contained four action classes. Each action class had three evaluation levels: good, average, and bad. The raw action signals were resized to the same dimension by using bi-linear interpolation. Then, a three-layer LSTM followed by three fully connected layers and a softmax layer model was constructed to predict the category. The loss function $L_{p}$ is defined as (10):

(10) $$L_{p}=CrossEntropy\left(x,y\right).$$

The standard cross entropy error is used as the loss term, where *x* is the input signal and *y* is the ground truth label.

### 4.2. Condition Loss

Once the LSTM is trained, the weights of the last LSTM layer are used as a classifier in this section. By using a classifier and a specially designed condition loss, the feature representation of each class can be reversely learned. Such a feature is referred to as the generalize feature or best feature for each level of each action class. We denote feature as *f* ∈ *R^N^*, and *M* is the number of action classes and *L* is the number of levels. Therefore, there are a total of *P* = *M × L* classes. The dimension of classifier *C* is *N × P*, since the output is *P* classes and the feature dimension is *N*. The matrix form of general feature G is defined as *P × N*, where the general features for each class are stacked row by row. We define the score matrix as *S* ∈ *R^P^* *^×^* *^P^*. For the signal of action class *m, m =* 0, …, *M*−1 and level *l*, *l* = 0, …, *L*−1, the score matrix *S* can be defined as follows:

(11) $$S=\{\begin{array}{c} s_{t,t}=1,\;where\;t=m\cdot M+l\\ otherwise\;0 \end{array}.$$

It means for the signal of class *m* and level *l*, the entry *s*_*t*,*t*_ should be the largest (equals to one) among all other entries (equals to zero) in the same row. Then, we can define the condition loss *L*_c_ as follows:

(12) $$L_{c}=\|G\times C-S{\|}_{2}.$$

The objective is to minimize the distance of G * C and *S*, where *S* serves as the ground truth score. By minimizing the loss function, the signal of class *m* and level *l* should have the largest score in the entry *s*_*t*,*t*_. In one training iteration, when the training of the classifier is finished, *C* and *S* are fixed to find *G*. This forces the general feature to obtain the highest score in *S*.

### 4.3. Evaluation Loss

To calculate the evaluation score, we use the output of the last layer in the LSTM and the general feature discussed in Section 4.2 to get the cosine angle. The cosine angle is used to estimate the similarity of two vectors. The angle is normalized to the range 0 to 1. The formula is given by (13):

(13) $$score=\frac{-1}{\pi }\times Arccos\left(\frac{feature_{LSTM}\times feature_{Gen}^{T}}{\left\|feature_{LSTM}\|\times \|feature_{Gen}\right\|}\right)+1,$$

where $feature_{LSTM}$ is the feature extracted by the last layer in the LSTM and $feature_{Gen}$ is the general feature. In one training iteration, when the training of the classifier is finished and the general features are produced, the class of the input signal can be determined by the trained classifier. Since one class have three levels, the extracted feature is compared with three general features within the same class. We can obtain the maximum score from three evaluation scores for one action class at three different levels by the operator max(.). Then, it is multiplied by a scaling scaler *interval* to get the final evaluation:

(14) Evaluation=max(score)×interval.

If the interval is set to 100, the evaluation is scaled to the range 0 to 100. In order to use cross entropy to update the model, we transform the score into a label. We manually set equal ranges for each level. If the evaluation is inside the range, it will be marked as a label. We define the formula as (15):

(15) $$Evaluation\;label=\{\begin{array}{c} 0\;,\;subject\;Evaluation\;Score=\left\{x|0\leq x<33\right\}\\ 1\;,\;subject\;Evaluation\;Score=\left\{x|33\leq x<66\right\}\\ 2\;,\;subject\;Evaluation\;Score=\left\{x|66\leq x<100\right\} \end{array}.$$

Now, we have the evaluation label. Using evaluation label and ground truth $y_{true}$, the loss function *L_e_* can be defined by the following:

(16) $$L_{e}=CrossEntropy\left(y_{true},\;Evaluation\;label\right).$$

The total loss function we use to update our model is given by (17):

(17) $$L_{total}=\alpha L_{p}+\beta L_{c}+\gamma L_{e},$$

where α,β, and γ are the balancing parameters for three loss terms. $L_{p},\;L_{c},\;L_{e}$ are prediction loss, condition loss, and evaluation loss, respectively. After the model is trained, the output of the evaluation model is the evaluation score. The users may match the scores to the range defined by Equation (15) to see their performance at three levels: good, average, or bad.

## 5. Results

### 5.1. Dataset

We used a wearable human activity recognition folder (WHARF) dataset [35] and a rehabilitation action evaluation dataset which we collected for our experiments. WHARF contained 14 kinds of activity signals collected by triaxial accelerometer from daily life. The sample rate of the sensor data was 32 Hz. We used sliding window (size: 80, overlap: 50%) for preprocessing. In each class, we took 120 samples for training and the rest for testing. The number of states in each class was set to 6 for the S-CNN’s state dictionary. The part number was 4 for the D-CNN. All neural network models used the same parameter settings with learning rate 0.01, momentum 0.9, and weight decay 0.0005. We also used a Skoda checkpoint dataset [37] for our classification experiments. It contained 10 distinctive activities from a car maintenance scenario. In each class, we took 400 samples for training and the rest for testing.

Although the WHARF dataset was suitable for action recognition, it did not provide the ground truth of action evaluation. Since the scores of the action dataset are not common, we collected our own dataset by asking users to wear eight sensors—five on the shoulders and back, two on the elbows, and one on the forehead. The sensor devices transmitted action signals to the receiver. We used a DA14583 IoT (Dialog Semiconductor, Taipei, Taiwan) sensor development kit to collect our data. This sensor product is manufactured by the Dialog Semiconductor company. It consisted of a 12-DOF wireless sensor module with development platform. The sensor settings were 12.5 Hz Acc, 25 Hz Gyro, and 10 Hz Magneto. And the mobile app version is 3.250.12. Then, we used the signals to train our model and evaluate the results.

**Figure 6:**
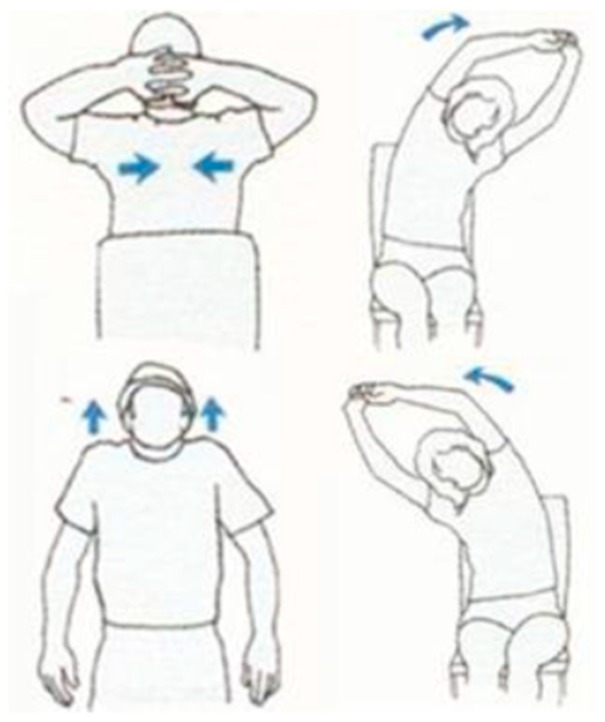
Four types of rehabilitation exercises.

In our rehabilitation action evaluation dataset, we found 49 healthy volunteers to collect the dataset. First, we had a total of four actions and each action had three different levels (i.e., good, average, and bad). These actions and levels were defined by the rehabilitation physicians. The dataset contained twelve exercise classes in total. Second, each level of each action had to be performed ten times in a row. Third, we took a guided film as a demonstration to ensure the quality of the collected data. Volunteers were only required to follow the actions of the person in the film. In addition, we gave clear instructions to remind the volunteers of the next movement. The four actions are depicted in Figure 6. In the first row, the first exercise contains three actions: raise both hands and put the hands in the back of the head with ten fingers crossed. Then, push the elbows back to the body. The second/last exercise contains two actions: stretch both hands up with ten fingers crossed, and then bend over to the left/right. In the second row, the first exercise contains one action: lift both shoulders up. Since each action is performed ten times in a row, we had to separate the data one by one. Autocorrelation was applied to find a single action. The autocorrelation is given by Equation (18):

(18) $$\mathrm{AutoCorrelation}\left(X,k\right)=\frac{\frac{1}{T}\sum_{t=1}^{T}\left(X_{t}-\bar{X}\right)\left(X_{t+k}-\bar{X}\right)}{\frac{1}{T}\sum_{t=1}^{T}{\left(X_{t}-\bar{X}\right)}^{2}},$$

where *X* is the original action signal, *T* is the time length of the action signal, and *k* is the field. We also found all peaks of the self-correlation graph and considered them as candidates.

(19) PKS(X)=FindPeaks(AutoCorrelation(X, k))

When there are more than ten candidates, we calculated the distance between each candidate and removed candidates with a distance below a predefined threshold. The threshold can be determined by calculating the average distance between candidates. Cut point $\hat{x}$ is the point to split the action signal by calculating the variance. The formulation is defined by (20):

(20) $$\hat{x}=\underset{\left(x_{1},x_{2},\ldots ,x_{10}\right)}{\mathrm{argmin}}Var\left(E_{x_{i}\sim PKS\left(X\right),x_{j}\sim PKS\left(X\right)}\left[\|x_{i}-x_{j}{\|}_{2}\right]\right)$$

**Figure 7:**
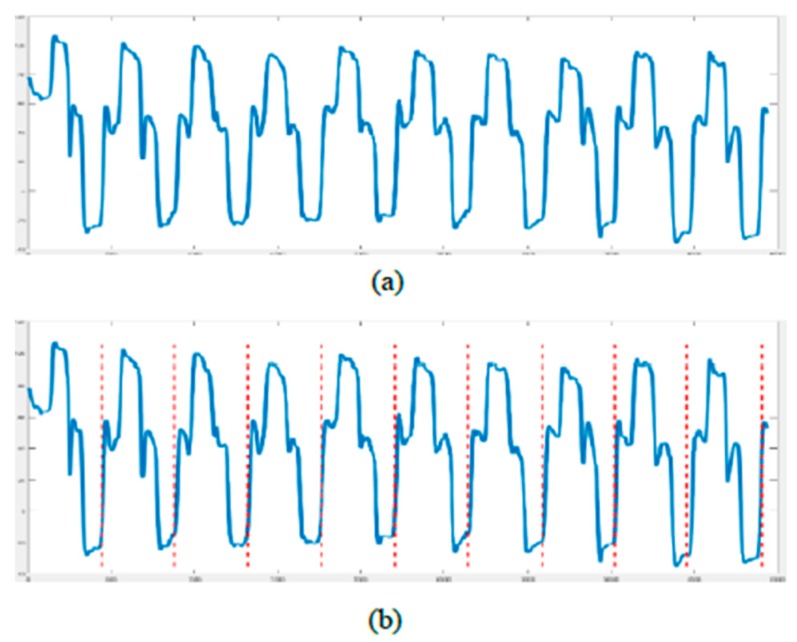
(**a**) Original signals. (**b**) Signals after partitioning.

The original action signals are depicted in Figure 7a. There are ten peaks in this graph. Figure 7b shows the signals after data preprocessing to find ten segments.

### 5.2. Experimental Results of the MP-CNN

**Figure 8:**
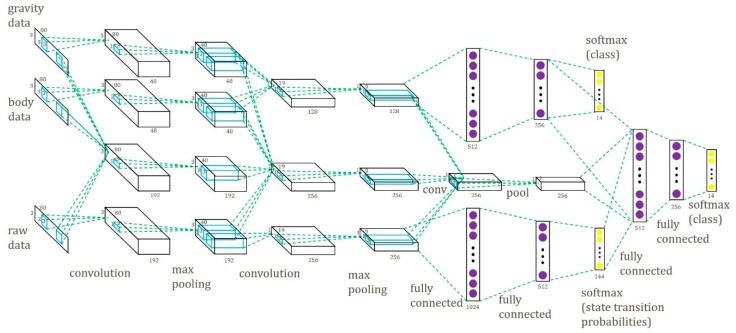
MP-CNN-1(1,2,3) model.

To evaluate the performance of the proposed MP-CNN, two types of MP-CNN were used. In the following experiments, MP-CNN-1 indicates that the GB-CNN is used in the top part of the MP-CNN, whereas the S-CNN is used in the bottom part. MP-CNN-2 indicates that the GB-CNN with the D-CNN are used in the top part of the MP-CNN, whereas the S-CNN is used in the bottom part. In the MP-CNN, the middle path captures the correlation between the top and bottom models. Different numbers of convolution and pooling layers are evaluated in the middle path in the MP-CNN model. The MP-CNN-1(1,2,3) means that there are three convolution operations and pooling layers in the middle path corresponding to the results from the top and bottom models. It is depicted in Figure 8. The MP-CNN-1(2,3) applies convolution and pooling twice in the middle path. The MP-CNN-1(3) and MP-CNN-2(3) do convolution and pooling once in the middle path, shown in Figure 3 and Figure 4, respectively. The MP-CNN-1(0) and MP-CNN-2(0) do not use the middle path. The results of the classification accuracy are presented in Table 1.

To evaluate the performance of the model combination, we compared a single CNN model to a combined model, including MP-CNN-1 (composed of a GB-CNN and an S-CNN) and MP-CNN-2 (composed of a D-CNN and an S-CNN). The single CNN model included a GB-CNN (top of Figure 3), an S-CNN (bottom of Figure 3), or a D-CNN (top of Figure 4). According to the results in Table 2, combining two CNN models can increase the accuracy.

We also compared the MP-CNN to other learning methods, including SVM, K-nearest neighbor (KNN), neural network (NN), GMM [35], signal image (SI) [38], and activity image (AI) [38]. The three-axis signals were concatenated into a single vector as input to train the SVM and used for KNN and NN. The NN had two hidden layers, which contained 1024 and 512 nodes, respectively. From [38], we used the three-axis signals of gravity, body, and raw signals to build a signal image (SI) and apply discrete Fourier transform (DFT) on SI to get an activity image (AI). Then, we used SI and AI as input data for the CNN model in [35]. According to the results in Table 3 and Table 4, MP-CNN-2 achieves the highest accuracy.

### 5.3. Action Evaluation Results

Our model architecture was based on LSTM. We also used other deep learning architectures for comparison. As shown in Table 5, ConvLSTM is the combination of CNN and LSTM. One can find more details in [42]. VGG [43] and ResNet [28] were the best models on ImageNet in 2014 and 2015. When VGG and ResNet are used as classifiers, the input signals are considered as 1-D vectors and 1-D convolutions are applied.

We also tried different units of LSTM, and found that 224 units achieved the best accuracy. Therefore, we set the dimensions of three LSTM layers as 224, 224, and 196. The results are presented in Table 6. In order to determine the proportion of training and testing, we conducted different experiments. In Table 7, subject_8 means that we selected eight people as training and others as testing. In our experiments, the selection of 36 people (contains 576 actions) as training and 13 people (contains 208 actions) as testing achieved the highest accuracy.

**Figure 9:**
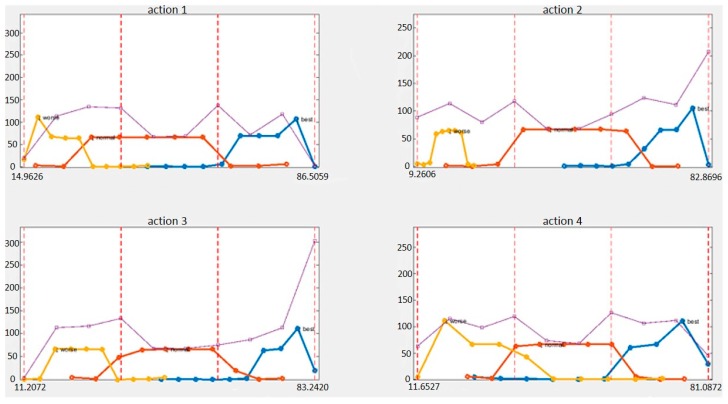
Score distribution of all actions for all levels.

Table 8 presents the results for precision, recall, and F1-Measure. Figure 9 shows the distribution of predicted evaluation scores for all levels for one action. The yellow line is a bad level, the red line is an average level, and the blue line is a good level. The purple line is the total of the three levels. 

## 6. Discussion

### 6.1. Discussion about the MP-CNN Results

Considering different numbers of layers in the middle path, we note that MP-CNN-1(3) and MP-CNN-2(3) had a higher classification accuracy (Table 1), which means that capturing the correlation of the output of the last pooling layer is sufficient. In addition, it also indicates that the classification accuracy is further improved when the D-CNN model is included in the framework. We can see that the MP-CNN achieves the highest accuracy when combining the D-CNN and the S-CNN (Table 2). As shown in Table 3 and Table 4, we can see that our model obtains the best classification accuracy on both the WHARF and the Skoda datasets.

### 6.2. Discussion about the Action Evaluation Results

Examining the experimental results for our action evaluation dataset, we note that the proposed model achieved a test accuracy of 90.63% (Table 5). It means that our action evaluation system can achieve a better performance than others. We also tried different dimension reductions of LSTM and different numbers of training and testing data. Our results show that more dimension reductions of LSTM and more training subjects can achieve a better performance. We also considered the accuracy of precision, recall and F1-Measure of our system. Action 9 had the worst F1-measure score (0.85 in Table 8). Finally, we showed the score distribution of all actions for all levels. Using the proposed evaluation architecture, the good and the bad levels are very separable. We note that, in all four actions, the highest score is 86.5059 and the worst score is 9.2606, which means that no matter how poorly you perform the rehabilitation, you will obtain 9 points. If the physical therapist performs the standard rehabilitation action, 86 points is obtained instead of 100 points. Overall, the scores of the three levels are reasonably distributed. Users can correct their actions based on these scores, and the model has the potential for practical applications.

## 7. Conclusions

A multipath convolutional neural network (MP-CNN) and an action evaluation method are proposed in this paper. An MP-CNN consists of a D-CNN and as S-CNN model. A D-CNN can assign similar signal segments to the same CNN channel. Therefore, it can better deal with the problem of data noises, data alignment, and other data variations. An S-CNN uses a modified LZW algorithm to extract the hidden state transition probabilities of raw data as discriminate features. Demonstrated by the experiments, the results of classification accuracy have shown that an MP-CNN is very effective for activity recognition using sensor data. Compared to recent machine learning-based methods, they are either required to train strong ensemble classifiers or powerful hand-crafted feature representations to achieve a high recognition accuracy. Deep learning-based action recognition discovers representative features and trains the classifier within an end-to-end model. In the future, we will extend our framework to complex activities and signals containing multiple activities. Moreover, we will design a model which can help users perform standard rehabilitation actions without a physical therapist. Our model provides an evaluation score that allows users to correct their actions based on that score. We propose a method to predict a score by using a classifier and a general feature. The experimental results also prove that the score obtained will be in the correct interval. In our future work, we will collect more data that contain more actions, and each action will contain more modes. Our research work will focus on unsupervised or action recognition as a new direction.

## Figures and Tables

**Table 1:** Accuracy of different numbers of middle layers in the MP-CNN.

**MP-CNN-1(1,2,3)**	**MP-CNN-1(2,3)**	**MP-CNN-1(3)**
77.87%	77.09%	78.52%
**MP-CNN-1(0)**	**MP-CNN-2(0)**	**MP-CNN-2(3)**
73.74%	75.55%	79.43%

**Table 2:** Accuracy of the single and combined CNN model.

**GB-CNN**	**S-CNN**	**D-CNN**
73.88%	69.64%	75.26%
**MP-CNN-1**	**MP-CNN-2**	
78.52%	79.43%	

**Table 3:** Classification accuracy of the WHARF dataset using different methods.

Method	Accuracy	Method	Accuracy
SVM	50.27%	SI [38]	66.51%
KNN	55.78%	AI [38]	71.45%
NN	57.71%	MP-CNN-1	78.52%
GMM	66.31%	MP-CNN-2	79.43%

**Table 4:** Classification accuracy of the Skoda dataset using different methods. We show our result in bold.

**KNN**	**SVM**	**NN**
87.67%	43.7%	74.99%
**SC [39]**	**PCNN [40]**	**AI [38]**
84.5%	88.19%	84.33%
**HMM-CNN [41]**	**MP-CNN-1**	**MP-CNN-2**
89.38%	**94.09%**	**94.69%**

**Table 5:** Accuracy (acc.) of our rehabilitation action dataset.

Architecture	Training acc.	Testing acc.
ConvLSTM [42]	99.64%	89.62%
VGG16 [43]	99.81%	90.13%
ResNet50 [28]	99.79%	90.25%
Proposed	100.00%	90.63%

**Table 6:** Accuracy of different dimension (dim.) reductions of long short-term memory (LSTM).

Feature Dim.	Epoch	Training acc.	Testing acc.
DIM_96	800	97.50%	84.65%
DIM_128	600	98.50%	86.85%
DIM_150	500	99.17%	87.69%
DIM_196	600	99.33%	88.64%
DIM_224	700	99.67%	98.73%

**Table 7:** Accuracy of different numbers of training and testing.

Dataset	Training acc.	Testing acc.
Subject_8	98.50%	86.65%
Subject_19	98.60%	90.23%
Subject_36	99.67%	90.63%

**Table 8:** Accuracy of precision, recall, and F1-Measure.

Action	Precision	Recall	F1-Measure	Support
0	0.87	0.95	0.91	65
1	0.88	0.85	0.86	71
2	0.90	0.93	0.92	76
3	0.91	0.94	0.92	65
4	0.94	0.92	0.93	65
5	0.92	0.92	0.92	66
6	0.86	0.92	0.89	62
7	0.91	0.92	0.92	76
8	0.91	0.82	0.86	61
9	0.90	0.81	0.85	68
10	0.91	0.88	0.89	56
11	0.84	0.88	0.86	59
Average	0.90	0.90	0.90	790
